# Predicting reproductive-phase nutrient replenishment in *Cannabis sativa* L. via a transpiration-driven mass-balance framework

**DOI:** 10.3389/fpls.2026.1955837

**Published:** 2026-10-12

**Authors:** Kit Powell, William L. Bauerle

**Affiliations:** Department of Horticulture and Landscape Architecture, Colorado State University, Fort Collins, CO, United States

**Keywords:** hemp, hydroponics, net nutrient removal, nutrient use, water use efficiency

## Abstract

Fixed nutrient recipes often overlook developmental shifts in uptake and the coupling between transpiration and ion delivery. We tested a transpiration-driven mass-balance framework to predict nutrient replenishment during the reproductive phase in two cultivars of *Cannabis sativa* L. (CJ2, First Light). Plants received a solution prepared from a constant recipe; water use was tracked gravimetrically, leachate was periodically sampled, and organs were harvested for ICP–OES/flow injection analysis and biomass composite partitioning. Water-use efficiency (WUE) peaked at 4.5 g L^−1^ in CJ2 and 3.4 g L^−1^ in First Light, while solution drawdown of N, P, and K declined from early to late flowering. Leachate chemistry resolved cultivar- and stage-dependent transitions between net nutrient removal (net removal) from solution and net accumulation above input. In First Light, N shifted from 130 mg L^−1^ net removal to +109 mg L^−1^ enrichment and K from 192 mg L^−1^ net removal to +195 mg L^−1^ enrichment; in CJ2, N ranged from 90 mg L^−1^ net removal to +101 mg L^−1^ enrichment and K from 102 mg L^−1^ net removal to +195 mg L^−1^ enrichment. Phosphorus differences were smaller (First Light: 58 to 13 mg L^−1^; CJ2: 42 mg L^−1^ removal to +2 mg L^−1^ enrichment). Mg net removal was greater in First Light than CJ2 (37 vs. 16 mg L^−1^). Significant cultivar main effects were detected for P, K, Ca, and Fe (p ≤ 0.05), whereas significant date x cultivar interactions were detected for Mg and Mn (p ≤ 0.05). Across the four harvest intervals retained for mass-balance comparison, biomass-weighted predictors frequently produced the lowest absolute percent deviation (APD), although performance was element- and cultivar-dependent. Single-organ tissue element concentrations misrepresented whole-plant replenishment estimates: leaf Ca and Mg increased while leaf N declined, flowers and roots accumulated P, and stems and roots diverged in K. Predicted input concentrations derived from organ biomass-weighted tissue elemental content improved the alignment between replenishment estimates and measured net solution removal. Biomass-weighted means excluding floral tissue predicted most element replenishment amounts well and performed most consistently in CJ2, whereas biomass-weighted means including all organs more frequently minimized APD in First Light, with absolute percent deviations<10–13%. Collectively, these results indicate that reproductive-phase nutrition in *Cannabis sativa* L. is well represented by a flux-based mass-balance replenishment framework informed by organ biomass-weighted nutrient composition.

## 1 Introduction

Predominantly, nutrient solution concentrations are determined by fertilizer compositions rather than plant uptake dynamics or physiological demand, often relying on electrical conductivity (EC) proxies instead of ion-specific control (Fathidarehnijeh et al., 2023; Sulaiman et al., 2025). This can lead to excessive or imbalanced fertilization, which undermines precision and sustainability. The high production costs of controlled environments, combined with the economic value of *Cannabis sativa* L. (*C. sativa*), make this crop particularly susceptible to excessive fertilizer application (Cockson et al., 2020; Saloner and Bernstein, 2023, 2022). As fertilizer costs rise and mineral reserves become increasingly limited, aligning nutrient supply with actual plant demand is essential for both economic viability and sustainable resource use (Fuentes-Peñailillo et al., 2024).

Transpiration is a biologically regulated physical process that links water and nutrient fluxes. Because evaporative demand drives xylem mass flow, ions are delivered to organs at rates modulated by water-use efficiency (WUE), which links environmental conditions to transpiration-driven nutrient budgeting. A justifiable fertigation strategy is to replace water and ions removed by plants rather than maintain constant element setpoints within solution (Bugbee, 2004; Langenfeld et al., 2022; Langenfeld and Bugbee, 2024). This shifts nutrient management from static concentration control to dynamic flux replacement, a need that intensifies as nutrient uptake and partitioning change after floral initiation (Hershkowitz et al., 2025; Massuela et al., 2023; Westmoreland and Bugbee, 2022). Flux-based replacement inherently aligns fertigation with physiological demand and developmental dynamics, improving precision, sustainability, and the efficient use of increasingly scarce mineral and freshwater resources (Fuentes-Peñailillo et al., 2024; Hershkowitz et al., 2025).

Following floral initiation, biomass allocation in *C. sativa* shifts toward reproductive structures, lowering the root:shoot ratio as inflorescences expand (Saloner and Bernstein, 2023). This transition modifies nutrient demand and mobility: calcium uptake declines late in flowering despite ongoing floral growth, whereas phosphorus, potassium, and magnesium translocation increases (Saloner and Bernstein, 2023). These dynamics underscore the limitations of static nutrient recipes and the need for stage-specific, flux-based supply. Similar discrepancies between balance-derived uptake and tissue estimates occur in tomato for nitrogen, magnesium, and sulfur (Cedeño et al., 2024). Because mass flow governs delivery of ions with limited phloem mobility, environmental effects on WUE directly influence transport (Langenfeld et al., 2022; Massuela et al., 2023). Incorporating WUE into mass-balance calculations therefore provides a robust basis for adjusting nutrient supply across environments and developmental stages (Langenfeld et al., 2022; Massuela et al., 2023; Sperling et al., 2020).

Reproductive-phase fertigation in *C. sativa* remains insufficiently resolved because nutrient composition, WUE, and ion-uptake dynamics have rarely been integrated into a unified framework (Cedeño et al., 2024; Saloner and Bernstein, 2023; Westmoreland and Bugbee, 2022). Most studies emphasize single nutrient concentration targets rather than coupling optimal tissue elemental requirements to water-driven mass flow. The present work closes this gap by using a transpiration-driven mass-balance approach to quantify reproductive-stage nutrient depletion and/or accumulation in organ tissue and organ-specific biomass-weighted elemental composition measured water flux. This mechanistic framework links tissue element concentration and WUE directly to uptake-mediated depletion of nutrients and water from substrate solution, enabling the estimation of fertigation solution element replenishment rates that are constrained by both developmental shifts in nutrient uptake and partitioning and the biophysics of transpiration-driven xylem transport.

Accordingly, this study investigates: (i) how organ-level nutrient concentrations and whole-plant nutrient biomass-weighted elemental composition relate to changes in solution nutrient concentrations; (ii) how WUE adjusts nutrient concentrations in irrigation solutions to meet tissue-based requirements across environments, cultivars, and developmental stages; and (iii) the extent to which leachate element composition corroborates or contradicts mass balance estimates of water removal and nutrient input replenishment concentrations. By integrating organ-weighted tissue element data with WUE, this work provides a reproducible procedure for calculating solution input replenishment recipes, defining cultivar-specific nutrient uptake profiles, and establishing a framework to distinguish reproductive-phase changes in organ- and whole-plant level nutrient requirements.

## 2 Materials and methods

### 2.1 Plant material and clonal propagation

Ninety cuttings (45 per cultivar) of two *C. sativa* cultivars, CJ2 and First Light, were excised and clonally propagated. On 9, August 2024, 1-inch rockwool cubes (Grodan, Milton, Ontario, Canada) were presoaked in deionized water (DI) containing a diluted complete fertilizer solution (12-2–12 Plug Special, Plant Marvel Chicago Heights, Illinois, USA) adjusted to an electrical conductivity (EC) of 0.3 dS·m^−1^ and pH 6.0. Each cutting was prepared by making a diagonal basal incision and dipping the base into a rooting gel containing indole-3-butyric acid (Clonex, Growth Technology Ltd., Taunton, Somerset, UK) before insertion into the rockwool. Leaves on each cutting were trimmed to approximately 50% of their original area to limit transpiration during rooting.

Cuttings were placed in 1020 trays in a growth chamber (Conviron, Model PG2500, Pembina, North Dakota, USA). Chamber environmental conditions were 24 h photoperiod at 340 µmol·m^−2^·s^−1^ photosynthetic photon flux density (PPFD), 22 °C air temperature, and 80% relative humidity (RH). The cubes were remoistened periodically with the fertilizer solution. After 10 days, plants were moved to a research bay at the Colorado State University Horticulture Center. Environmental conditions of the bay were controlled via venting, forced air, and an end wall evaporative cooling system to maintain an air temperature set point of 26.5 °C, and natural gas heating set to initiate at 18.3 °C. Vapor pressure deficit (VPD) varied throughout the experiment, with the majority of daily mean values between 1.2 and 1.6 kPa. Supplemental lighting was initially set to an 18/6-h (day/night) photoperiod based on vegetative industry standards and supported by literature by (Ahrens et al., 2023). Roots emerged after one week. After 18 days from excision, 23 vigorous plants per cultivar were chosen from the initial 45 cuttings per cultivar for growth uniformity and transplanted into 4-inch rockwool cubes (Growdan, Milton, Ontario, Canada) pre-soaked in a complete fertilizer solution (EC 1.0 dS·m^−1^, pH 6.0). On September 1, these planted 4-inch cubes, 46 in total (23 per cultivar), were seated into pre-positioned 11-L Bato buckets (CropKing Inc., Lodi, Ohio, USA) filled with 1.5 kg oven dry horticultural perlite (Therm-O-Rock, course grade horticultural perlite, Chandler, AZ), a substrate with low cation-exchange capacity (CEC). We note that low CEC does not exclude nutrient retention or release.

### 2.2 Greenhouse experimental design and fertigation

Fertigation was delivered via two dripper stakes at a rate of 1.9 liter·min^−1^. Essential elements were delivered via calibrated pneumatic pistons as described in (Powell and Bauerle, 2026). The nutrient input solution was formulated as a non-limiting diagnostic solution rather than a crop-specific production recipe, with elemental concentrations maintained above anticipated demand to enable quantification of net solution removal, enrichment above input, and ion-specific temporal dynamics. The recipe concentrations were as follows: nitrogen [N] (nitrate – 250 ppm, ammonium - 6.3 ppm)), potassium [K] – 350 ppm, phosphorus [P] – 60 ppm, magnesium [Mg] – 61.7 ppm, calcium [Ca] – 100 ppm, iron [Fe] – 2 ppm, zinc [Zn] – 0.23 ppm, boron [B] – 0.09 ppm, copper [Cu] – 0.23 ppm, molybdenum [Mo] – 0.07 ppm, and manganese [Mn] – 0.27 ppm. We note that before the reproductive-phase experiment began in August 2024, Bruce Bugbee discussed preliminary findings on P supply in cannabis with us on 7 August 2024 (B. Bugbee, personal communication). On that basis, we increased the P concentration of the input nutrient solution by 10 mg L^−1^, from 50 to 60 mg L^−1^, relative to the earlier vegetative-phase experiment. The findings discussed at that time were subsequently published by Hershkowitz et al. (2025). Initially, fertigation was supplied four times daily (0700, 1100, 1500, and 2000 h). For the primary fertigation cycles, the duration was determined based on observed leachate at each fertigation event to ensure substrate saturation. The schedule was subsequently changed to two daily events (0700 and 2000 h). The interval between the two daily fertigation events was treated as a semi-closed mass-balance period, uninterrupted by additional nutrient inputs, during which changes in solution composition reflected net nutrient partitioning among plant removal, root-zone/substrate retention, and the remaining container solution. To minimize substrate evaporative water loss, the surface of each container was covered with white plastic. To initiate flowering, supplemental lighting was switched on 10, September 2024 to a 12/12 photoperiod, a photoperiod known to change C. *sativa* from the vegetative to the reproductive phase. In addition, black plastic was hung on the greenhouse wall adjacent to the research bay to prevent light pollution. Natural day length at this date was approximately 12 h 40 min (National Oceanic and Atmospheric Administration, n.d), which was less than the dark period length required for *C. sativa’s* reproductive phase (Ahrens et al., 2023).

### 2.3 Continuous gravimetric water-use measurements

Of the 23 replicate plants per cultivar, eight random replicates were placed on independent gravimetric balances (Adam Equipment GBK-35A Milton Keynes, Buckinghamshire UK). The plants were positioned in a 5 x 8 matrix with 1.5 m inter-plant spacing. Each balance interfaced with a custom IoT data-acquisition system (Adafruit ESP32 Feather, lithium-battery powered) via RS-232. Mass measurements were transmitted every 10 min to a ThingSpeak dashboard for real-time visualization and data storage (MathWorks, n.d).

The fertilizer injector and specified recipe were used to prepare a batch of nutrient solution sufficient to supply all plants for that day. The prepared batch was sampled to determine the measured base-solution nutrient concentrations used in the calculations. Net solution removal was calculated as the measured concentration in the corresponding base solution minus the measured pour through leachate concentration. Prior to substrate leachate extraction, the substrate was watered to container capacity and allowed to drain for 30 minutes (we note that container capacity alone does not establish air-filled porosity). Then, water transpired, was recorded throughout a 12 h-day course. After 12 h, the quantity of solution transpired was replaced with an equal amount of DI to return the substrate to container capacity. Resupplied DI was permitted to stabilize for 25 minutes. Thereafter, following best practice pour through procedures described in Altland (2021), an additional mass of DI displacement solution was added to the substrate surface to hydraulically push 230 ml of solution out of the drainage elbow. During drainage, samples were filtered through 18×16 mesh screen, and the first 230 ml of solution was collected in clean, unused bottles. We note that the amount of displacement solution collected (approximately 2% of container volume) was predetermined to provide a sample that represents the entire bulk of the substrate, not just the bottom layer (Altland, 2021). Samples were immediately transferred to refrigerated storage for later analysis. Leachate sampling was conducted every tenth day for the duration of the reproductive phase (September 10 – November 9, 2024), yielding seven closed-system sampling events of eight replicate leachate samples per cultivar throughout the reproductive growth phase. Input-solution batch and leachate concentrations were quantified by Brookside Laboratories (New Bremen, OH, USA). NO_3_-N and NH_4_-N concentrations were quantified separately by flow injection analysis (FIA; FIAlyzer-2000, FIAlab) and summed to represent inorganic N in the nutrient-solution mass-balance calculations. Nitrate-N in the nutrient solution was correspondingly analyzed using cadmium reduction [method W-1.80 in Miller et al. (2013)]. Soluble B in nutrient-solution and leachate samples was measured by inductively coupled plasma–optical emission spectrometry (ICP-OES; iCAP PRO Duo, Thermo Fisher Scientific, Waltham, MA, USA) following method W-1.50 of Miller et al. (2013). The published method detection limit for B is approximately 0.10 mg L^−1^. Boron concentrations are reported to the nearest 0.01 mg L^−1^, consistent with Miller et al. (2013, p. 138). Soluble P, K, Ca, Mg, Fe, Cu, Mn, and Zn were measured using the same instrument following method W-2.00 of Miller et al. (2013). For this method, Miller et al. (2013, p. 147) report a detection limit of 0.05 mg L^−1^ and state that results are generally reproducible within ± 7.0%. Concentrations of these elements are reported to two significant figures, consistent with the reporting convention specified in that method.

### 2.4 Plant uniformity measurements

At 10-day intervals, morphological data were recorded for each of the 23 plants per cultivar. Stem caliper was measured at a consistent position 5 cm above the rockwool cube; two perpendicular readings (x and y) were averaged. Plant height was measured from the rim of the Bato bucket to the apex of the main stem. Canopy spread was quantified as the mean of orthogonal canopy diameters transversing the center perpendicular to one another.

### 2.5 Biomass and organ tissue analysis

In conjunction with leachate sampling, destructive harvests were performed at 10-day intervals beginning September 10, 2024, with the harvest of 6 plants (3 per cultivar). Plants were partitioned into leaves (defoliated), stems, roots (washed), and flowers (n = 3 biological replicates per cultivar per harvest). At the first harvest following the photoperiod change to a 12/12 cycle (0 days post photoperiod change; DPP 0), and again at the subsequent harvest (DPP 10), inflorescences had not yet developed sufficient biomass for reliable quantification. We note that at the time of each harvest the sugar leaves, floral peduncle leaves per inflorescence, were excised then pooled along with the defoliated leaves Leaf area was measured using a leaf area meter (LI-COR LI-3100, Lincoln, NE, USA). All tissues were oven-dried at 70 °C for ≥5 days to constant mass, then dry weights were recorded.

Organ specific tissue was masticated per replicate per cultivar using a stainless-steel grinder. Samples (5g) were then collected from each replicate organ for an overall organ representation per plant and sent for analysis. Organ-specific elemental concentrations were quantified by Brookside Laboratories (New Bremen, OH, USA). Tissue P, K, Ca, Mg, Fe, Cu, Mn, B, and Zn were quantified by ICP-OES (Thermo iCAP PRO Duo), whereas tissue N was reported as total nitrogen on a dry-weight basis. Nitrate-N was measured by digesting tissue samples in 27 mM calcium hydroxide and analyzing them with the cadmium reduction method for flow injection analysis (QuikChem Method 12-107-04-1-C). Dry tissue samples were analyzed for total N using combustion [method B-2.20 in Miller et al. (2013)].

### 2.6 Nutrient solution input concentration estimation via mass balance

A nutrient mass-balance-based approach was used to estimate individual element solution replacement concentrations as described in Langenfeld et al. (2022). The nutrient mass balance procedure assumes that nutrients in a closed system are either in the nutrient solution or in the plant tissue. The predicted nutrient replacement input concentration for each essential nutrient is determined as the product of organ tissue element concentration and the rate at which nutrients are removed from solution i.e. the plant WUE (Langenfeld et al., 2022) where:

(1) $$C_{input}=C_{tissue}\;x\;WUE$$

As previously described, mean tissue nutrient concentration (*C_tissue_*) (%; mg kg^-1^) per organ was determined using the analytical methods described in Section 2.5 and used in combination with WUE (Equation 2) to estimate individual element replenishment input concentrations (*C_input_*) (mg L^-1^) within the nutrient solution.

WUE was calculated as:

(2) $$WUE=\;\frac{M_{Dry}}{V_{irrigation}}$$

where (*M_Dry_*) is total plant dry biomass (g plant^-1^) and (*V_irrigation_*) is the total irrigation volume transpired per plant (L plant^-1^).

To estimate the whole-plant nutrient replenishment input concentration, an organ biomass-weighted mean was calculated by weighting each organ’s nutrient concentration by its fractional contribution to total biomass and then summing across all organs using the following equation:

(3) $$C_{weighted}=\sum_{i=1}^{n}\left(C_{organ}\;X\;\frac{Biomass_{organ}}{Biomass_{total}}\right)$$

where (*C_weighted_*) (mg L^-1^) is the whole-plant biomass-weighted mean, (*C_organ_*) (mg L^-1^) represents the nutrient concentration measured in each individual organ (leaf, stem, root, or flower), (*Biomass_organ_*) (g) is the dry biomass of the organ, and (*Biomass_total_*) (g) is the total above and below-ground plant biomass. This approach assumes 100% mass balance where water and nutrients are only removed from solution via root uptake and the transpiration stream. Thus, it integrates organ-specific nutrient concentrations with their proportional contribution to total biomass for whole-plant level net nutrient replacement estimates. Thus, it provides a physiologically meaningful estimate of the whole-plants net nutrient and water removal from solution and the amount that is needed to replace it. In effect, organs with greater biomass exert more influence on the weighted value, allowing the calculation to more accurately represent the nutrient status of the entire plant rather than a single organ type.

### 2.7 Absolute percent deviation and predictor evaluation

Prediction accuracy of organ-specific and biomass-weighted nutrient input estimates was quantified using absolute percent deviation (APD; Equation 4). APD was calculated as:

(4) $$APD=\frac{P-U}{U}\;X\;100$$

where P represents the predicted nutrient input concentration (mg L^−1^) derived from organ-specific or biomass-weighted tissue elemental concentration scaled by plant water use efficiency (Equations 1–3), and U represents the measured difference in solution nutrient concentration (mg L^−1^), calculated as the irrigation input concentration minus the measured pour-through leachate concentration at each harvest interval. This concentration difference was not calculated from nutrient mass divided by substrate solution volume and therefore did not assume that substrate solution volume was equivalent to transpired volume.

APD was evaluated for each element (except B), cultivar, predictor type (leaf, stem, root, flower, biomass-weighted including all organs [W], and biomass-weighted excluding flower tissue [WNF]), and harvest date. For each element–cultivar–harvest combination, the predictor yielding the lowest APD was identified as the optimal predictor for that interval. These harvest-resolved APD outcomes were used to evaluate temporal shifts in predictor performance across reproductive development.

For life-cycle comparisons, APD values were averaged across four harvest intervals restricted to periods in which mass-balance assumptions were satisfied. The first harvest was excluded due to incomplete pairing of tissue and leachate measurements, and the final two harvests were excluded because increasing substrate nutrient accumulation violated steady-state mass-balance conditions. Mean APD across the retained interval (DPP; 10-40) was used to identify the most accurate predictor over the reproductive phase.

### 2.8 Statistical analysis

Nutrient removal from solution are reported for nine essential elements and were analyzed using linear mixed-effects models (LMMs) to account for repeated measures across time and cultivar. Net solution removal values were modeled as a function of collection date (fixed within-subject factor), cultivar (fixed between-subject factor), and their interaction, with replicate treated as a random intercept to capture plant-level variance. Replicate was included as a random intercept to account for correlation among repeated observations from the same experimental unit. In cases where the estimated random-effect variance was small but greater than zero, the random intercept was retained because it indicated residual within-replicate correlation. Additionally, date, cultivar, and date × cultivar effects were evaluated using Type III tests with Satterthwaite denominator degrees of freedom. Corresponding F statistics, numerator and denominator degrees of freedom, and p-values are reported in the supplementary statistical table (**Supplementary Table 1**). Cultivar comparisons within harvest were evaluated using estimated marginal means with Bonferroni adjustment across the seven harvest dates (**Supplementary Table 2**). Models were fit using the lmer function in the lme4 package. To assess robustness to outliers and leverage points—identified via Cook’s distance—each model was re-estimated using robust linear mixed models (RLMMs) via rlmer from the robustlmm package and were used as a sensitivity analyses. Residual diagnostics (Q–Q plots, residuals vs. leverage, and Cook’s distance) were used to confirm model assumptions. B solution-removal observations are presented descriptively, B inferential results are not reported because the method-specific quantification limit could not be verified.

Pairwise comparisons between cultivars across time points were performed using estimated marginal means (EMMs) via the emmeans package, with Bonferroni adjustment for multiple testing. Summary plots with standard error bars (± SE) were generated using ggplot2 to visualize temporal net nutrient removal trends and cultivar separation. All statistical analyses were conducted in R (R Core Team, 2025 version 4.3.2). Predicted replenishment and measured net solution removal were each derived from biological observations with associated sampling variability, uncertainty was propagated by resampling the underlying observations rather than treating their calculated means as fixed values (**Supplementary Table 3**).

## 3 Results

### 3.1 Macro/micronutrient removal

**Figure 1** illustrates the net nutrient removal from the container fertigation solution (net solution removal) of the two cultivars throughout the course of the generative phase for the major elements. Overall, first light exhibited greater net nutrient removal from the fertigation solution than CJ2 for most macronutrients during the reproductive phase (**Figure 1**). Initially, net solution removal increased in both cultivars from DPP 0 and reached a maximum at DPP 20 before declining throughout the remainder of the reproductive phase. Negative values indicate that nutrient accumulation in the substrate solution exceeded plant removal, resulting in net enrichment of the irrigation solution relative to the input solution, first evident after harvest 6 (DPP 50) and declining further at harvest 7 (DPP 60). Net solution removal differences between cultivars, although significant, varied by element. In First Light, N shifted from 130 mg L^−1^ net removal to +109 mg L^−1^ accumulation and K from 192 mg L^−1^ net removal to +195 mg L^−1^ enrichment; in CJ2, N ranged from 90 mg L^−1^ net removal to +101 mg L^−1^ enrichment and K from 102 mg L^−1^ net removal to +195 mg L^−1^ enrichment. Phosphorus excursions were smaller (First Light: 58 to 13 mg L^−1^; CJ2: 42 mg L^−1^ removal to +2 mg L^−1^ enrichment). Mg net removal was greater in First Light than CJ2 (37 vs. 16 mg L^−1^). At the last two collection points, the substrate showed accumulation of the respective elements which led to negative values (accumulation).

**Figure 1:**
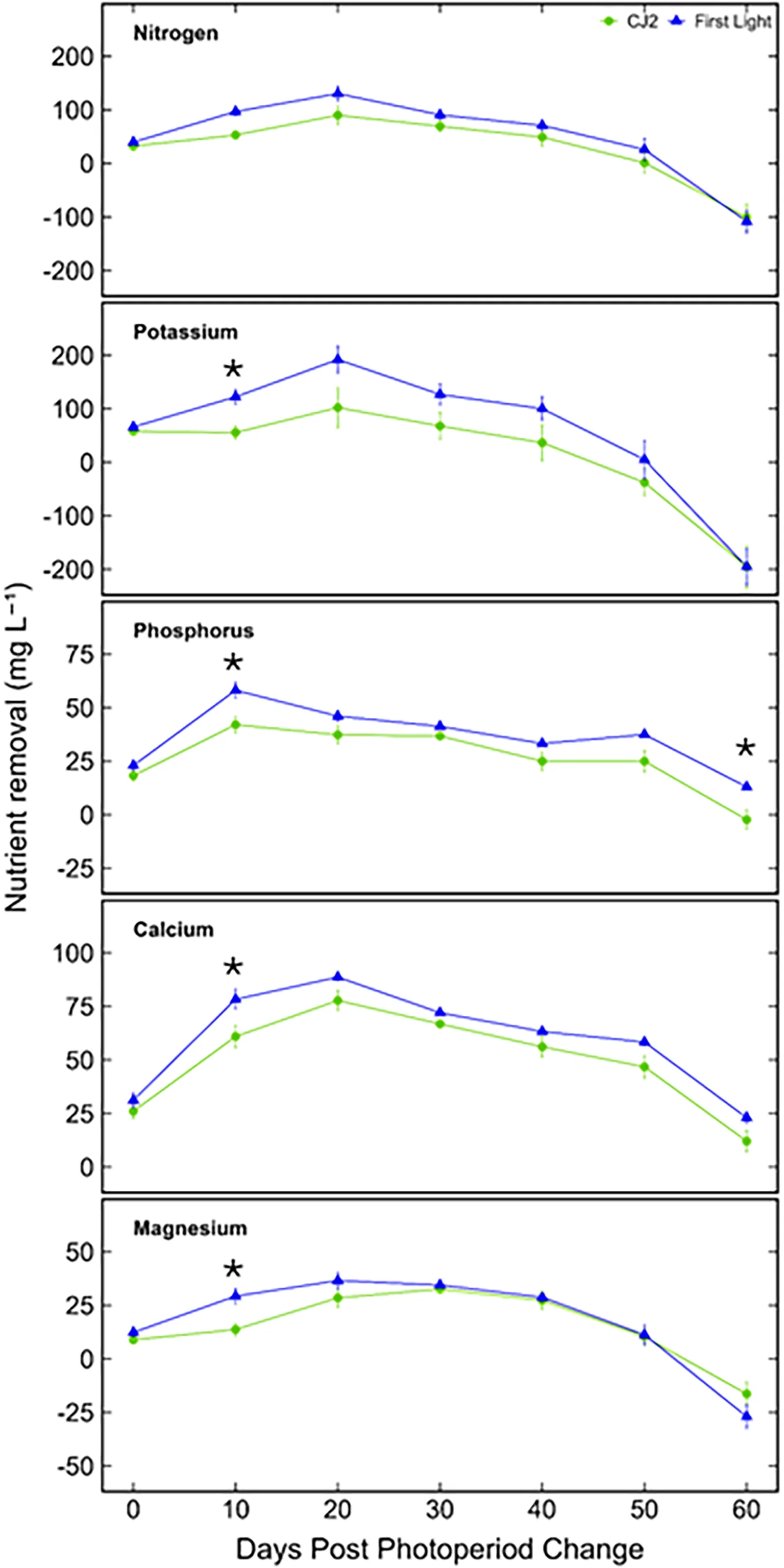
Calculated net removal of nitrogen, potassium, phosphorus, calcium, and magnesium from irrigation solution (mg L^−1^) from days following photoperiod change 0 – 60. Cultivar means for CJ2 (green solid circles) and First Light (blue solid triangles) ± SE (n = 8 per cultivar days following photoperiod change 0 – 40; n = 5 First Light and n = 4 CJ2 days following photoperiod change 50 and 60). An asterisk (*) indicates a statistically significant difference between cultivars (*P* < 0.05). Net removal is the measured input-solution base-batch concentration minus the measured leachate concentration. Note, SE bars are present for both cultivars but in some cases are small relative to the plotted symbols at some sampling dates and may be partially obscured.

Across the full reproductive phase, significant cultivar main effects were detected for P (p = 0.0036), K (p = 0.0319), and Ca (p = 0.0168), whereas N (p = 0.0597) and Mg (p = 0.2742) did not exhibit significant overall cultivar effects. For the micronutrients, Fe exhibited a significant cultivar main effect (p = 0.0125), whereas Cu (p = 0.1581), Mn (p = 0.0607), and Zn (p = 0.4799) did not. Significant date × cultivar interactions were detected for Mg (p = 0.0276), and Mn (p< 0.00001), indicating that cultivar differences for these elements varied across reproductive development.

During the first three leachate observation periods, several minor elements exhibited greater net solution removal in First Light than in CJ2, with the greatest separation occurring for Mn (**Figure 2**). Values exceeding the nominal recipe concentration reflect higher measured concentrations in individual input-solution batches. Net solution removal was calculated as the difference between measured input-solution and leachate concentrations. Boron results are presented descriptively throughout because low leachate B concentrations were below the method-specific limit of quantification, which exceeded 0.10 mg L^−1^. Calculated net B removal estimates increased from approximately 0.04 to 0.11 mg L^−1^ in both cultivars during early flowering, peaked at approximately 0.12 mg L^−1^, and declined after DPP 50. Manganese exhibited a significant date × cultivar interaction, with the greatest divergence occurring at DPP 0 and 10, when CJ2 increased from approximately 0.03 to 0.17 mg L^−1^ while First Light remained comparatively stable. Fe generally exhibited greater net solution removal in First Light during much of the reproductive phase, consistent with the significant overall cultivar effect (p = 0.0125). Zn and Cu showed no significant overall cultivar effects and no consistent cultivar separation throughout the reproductive phase.

**Figure 2:**
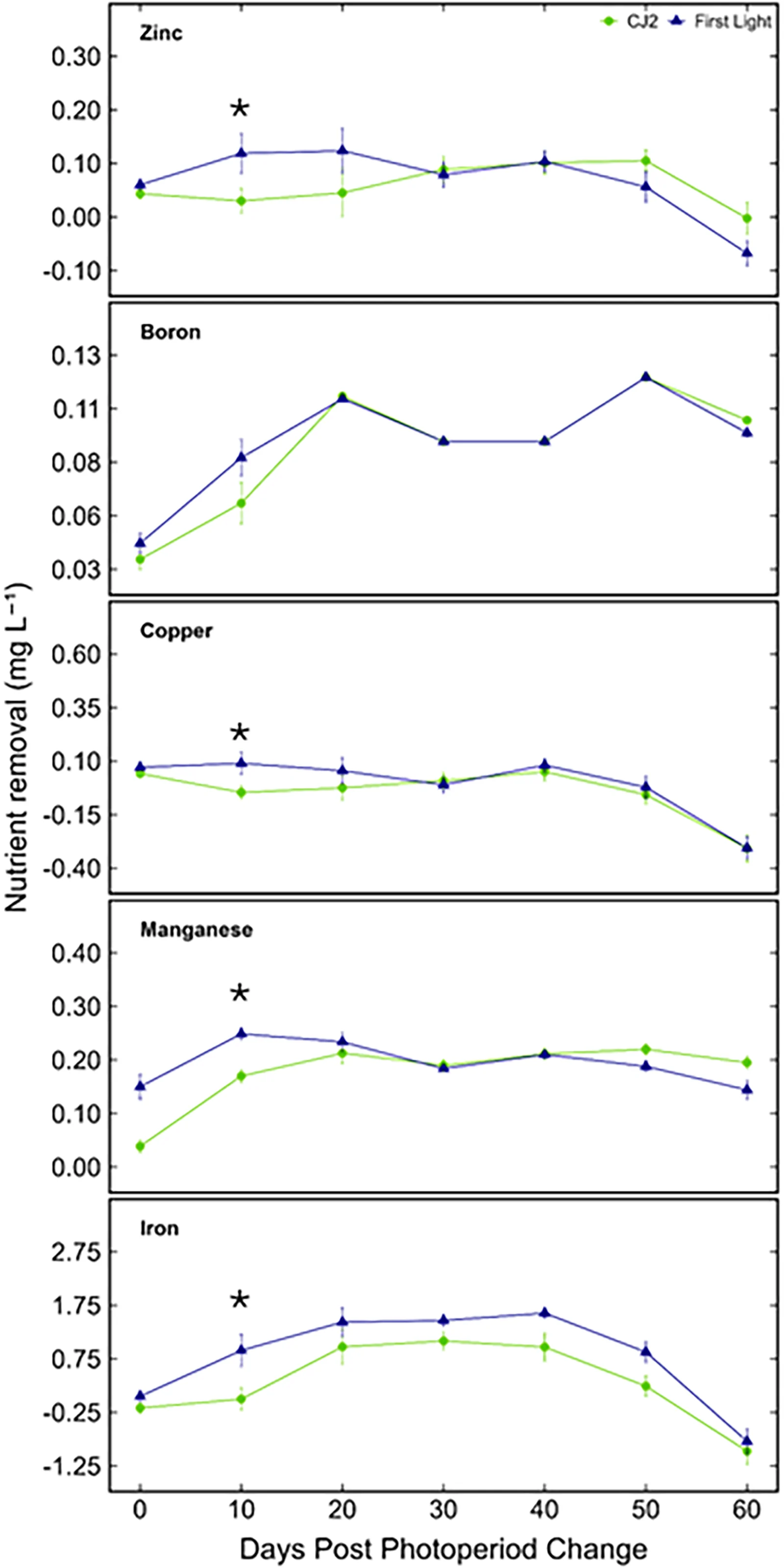
Calculated net removal of boron, copper, iron, manganese, and zinc from irrigation solution (mg L^−1^) from days following photoperiod change 0 – 60. Cultivar means for CJ2 (green solid circles) and First Light (blue solid triangles) ± SE (n = 8 per cultivar days following photoperiod change 0 – 40; n = 5 First Light and n = 4 CJ2 days following photoperiod change 50 and 60). For elements other than B, an asterisk (*) indicates a statistically significant difference between cultivars (*P* < 0.05). Net removal is the measured input-solution base-batch concentration minus the measured leachate concentration. Solution-derived B values are presented for descriptive purposes only; no inferences about statistical significance are made. Note, SE bars are present for both cultivars but in some cases are small relative to the plotted symbols at some sampling dates and may be partially obscured.

### 3.2 Water use efficiency

WUE was consistently greater in CJ2 than in First Light throughout the reproductive phase (**Figure 3**). CJ2 reached a maximum WUE of 4.50 g L^−1^ at DPP 30, whereas First Light exhibited a plateau at DPP 20 and 30 with WUE values of 3.39 and 3.33 g L^−1^, respectively. Despite these differences in magnitude, both cultivars exhibited similar temporal patterns, with WUE increasing and decreasing concurrently throughout the reproductive phase.

**Figure 3:**
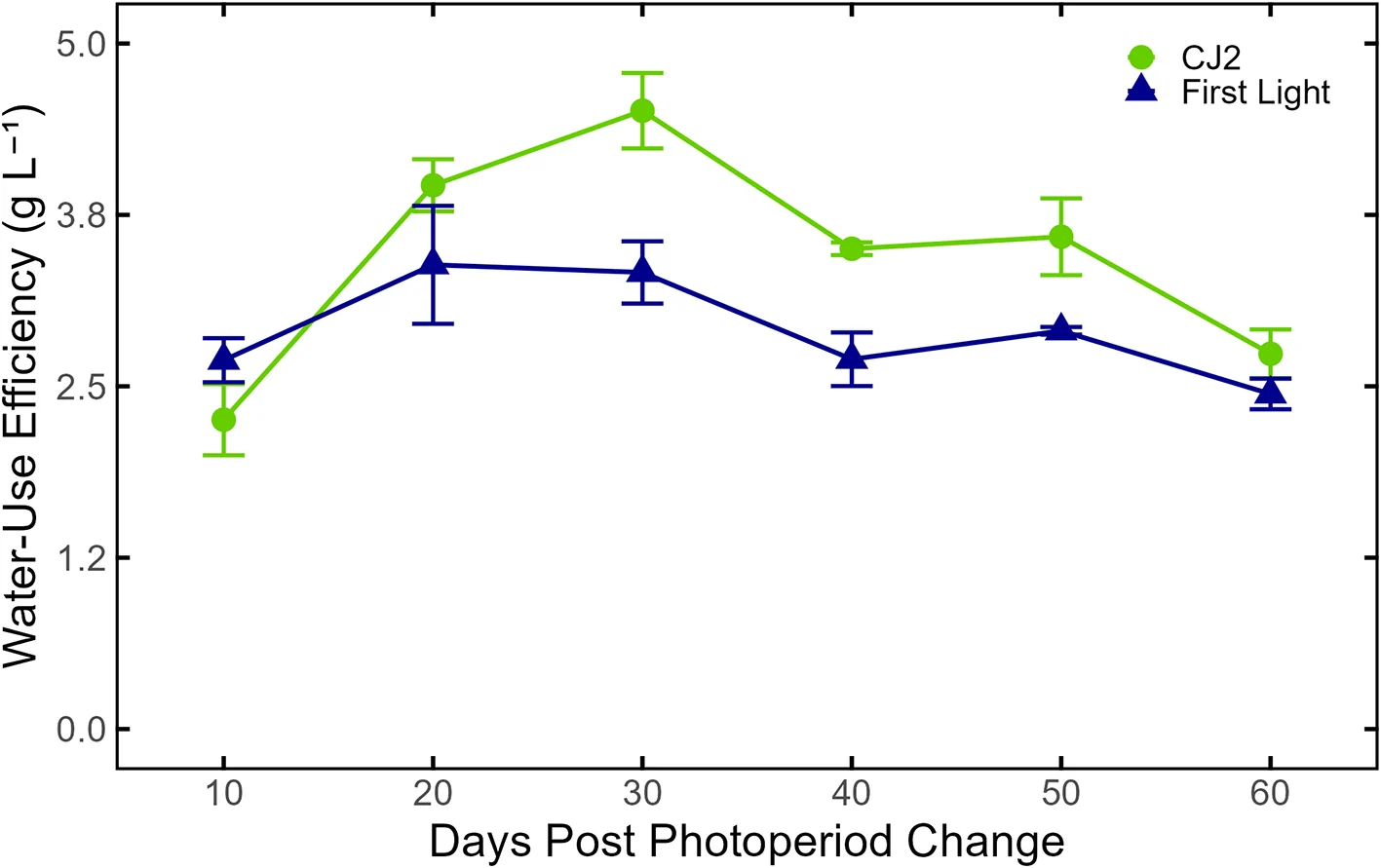
Water use efficiency (WUE, g L^−1^) throughout the study (days following photoperiod change 10 – 60) for the means of two cultivars First Light (blue solid triangle) and CJ2 (green solid circle) ± SE (n = 8).

### 3.3 Macro/micro tissue analysis

**Figure 4** illustrates the tissue element concentrations per organ over the course of the generative phase for the major elements. Ca and Mg concentrations steadily increased in leaf tissue, whereas N declined, and P and K stayed relatively constant. Similarly, P concentrations increased in CJ2 root tissue, whereas a decrease in Mg occurred in the root tissue of both cultivars (**Figure 4**). K exhibited a steady decrease in stem tissue concentrations, whereas other organ K concentrations remained relatively constant. Overall, macroelement leaf tissue nutrient concentrations were within sufficient ranges throughout the generative phase (Cockson et al., 2020; Hershkowitz et al., 2025; Landis et al., 2019).

**Figure 4:**
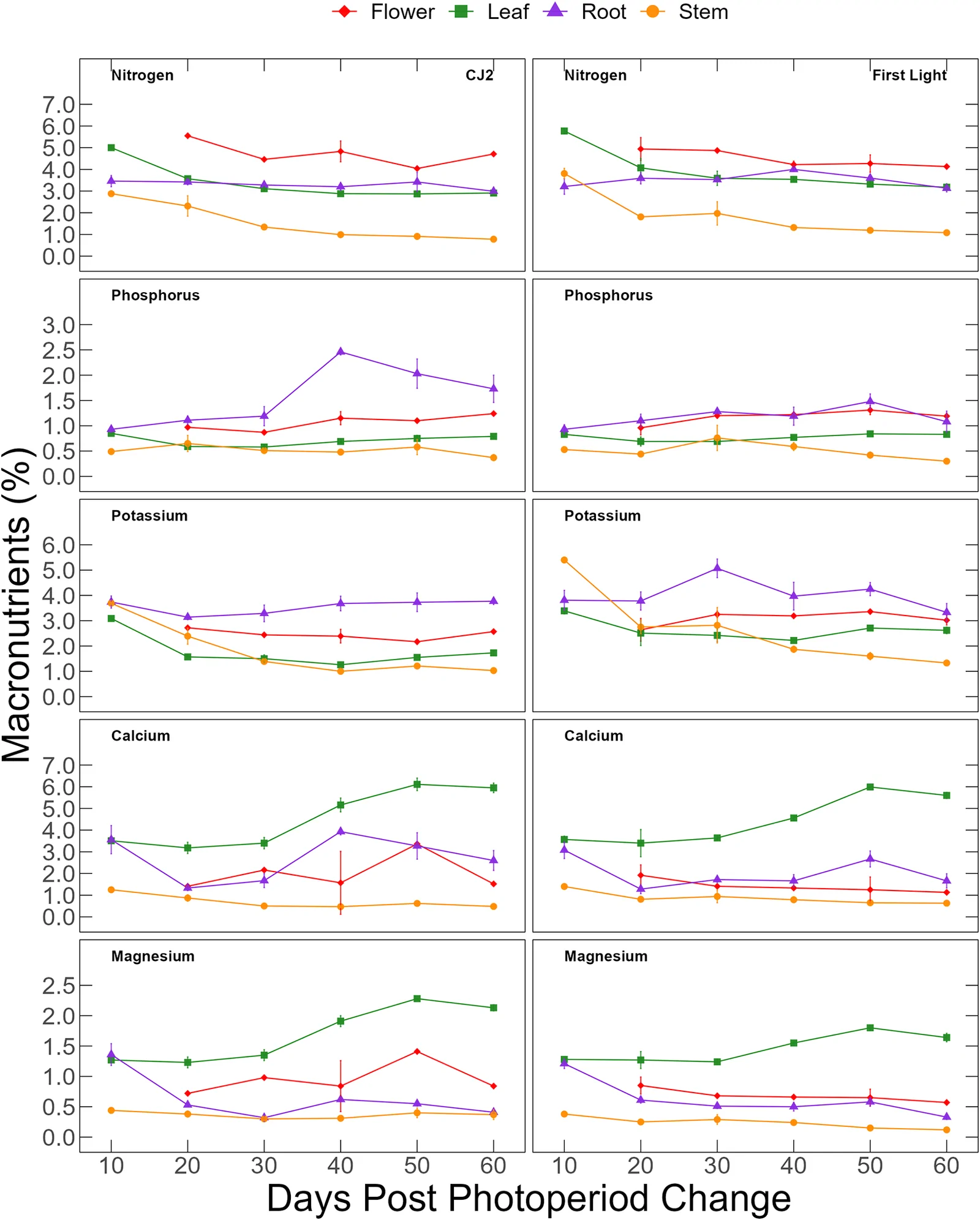
Tissue nutrient concentration (%) among organs for macro elements nitrogen, phosphorus, potassium, calcium, and magnesium from days following photoperiod change 0–60 for cultivars CJ2 and First Light. Color and symbols represent the following: (leaves = green solid square; flowers = red diamond; roots = purple triangle; and stem = orange circle) tissue concentrations. Symbols are the means of n = 3 replicates ± SE.

**Figure 5** illustrates the tissue element concentrations per organ over the course of the generative phase for the minor elements. Over the course of the reproductive phase, Cu root concentrations increased. Similarly, B leaf concentrations showed a steady increase, whereas all other organ Cu and B concentrations stayed relatively constant throughout the study (**Figure 5**). Initially, Fe and Mn were elevated in the root tissue but subsequently decreased to a relatively constant value thereafter. All other organ minor element tissue concentrations were relatively constant throughout the reproductive phase. Overall, minor element leaf tissue nutrient concentrations were within adequate ranges throughout the generative phase (Cockson et al., 2020; Hershkowitz et al., 2025; Landis et al., 2019).

**Figure 5:**
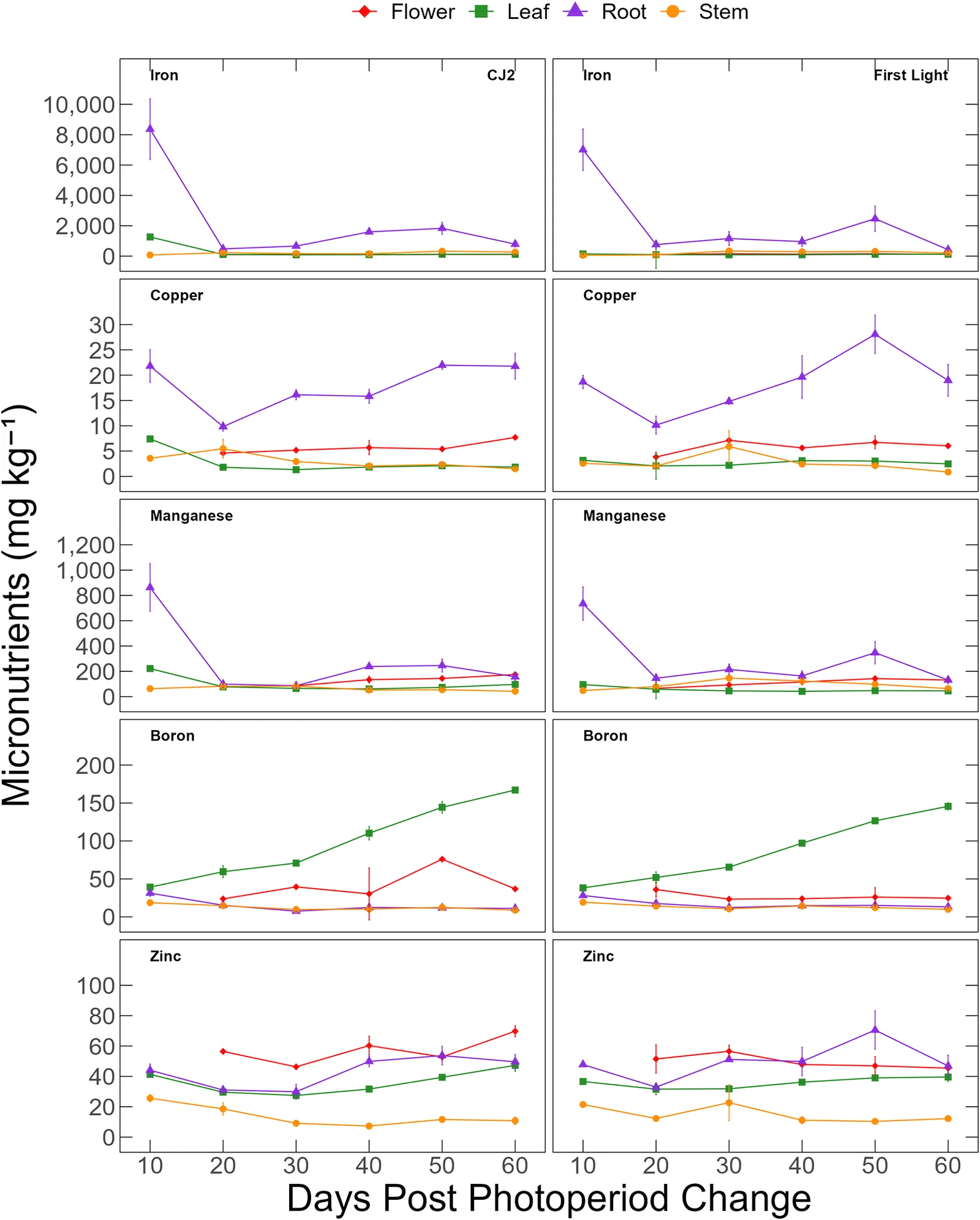
Tissue nutrient concentration (mg kg^−1^ dry weight (DW)) for micro elements iron, copper, manganese, boron, and zinc from days following photoperiod change 0–60 for cultivars CJ2 and First Light. Color and symbols represent the following: (leaves = green solid square; flowers = red diamond; roots = purple triangle; and stem = orange circle) tissue concentrations. Symbols are the means of n = 3 replicates ± SE.

### 3.4 Predicted net solution removal by organ

#### 3.4.1 Macronutrients

**Figure 6** illustrates the predicted nutrient replenishment concentrations for the macronutrients (N, P, K, Ca, and Mg) derived from organ-specific tissue concentrations compared with measured net solution removal from the irrigation solution. The predictor with the lowest observed APD was identified for each element (**Supplementary Table 4**).

**Figure 6:**
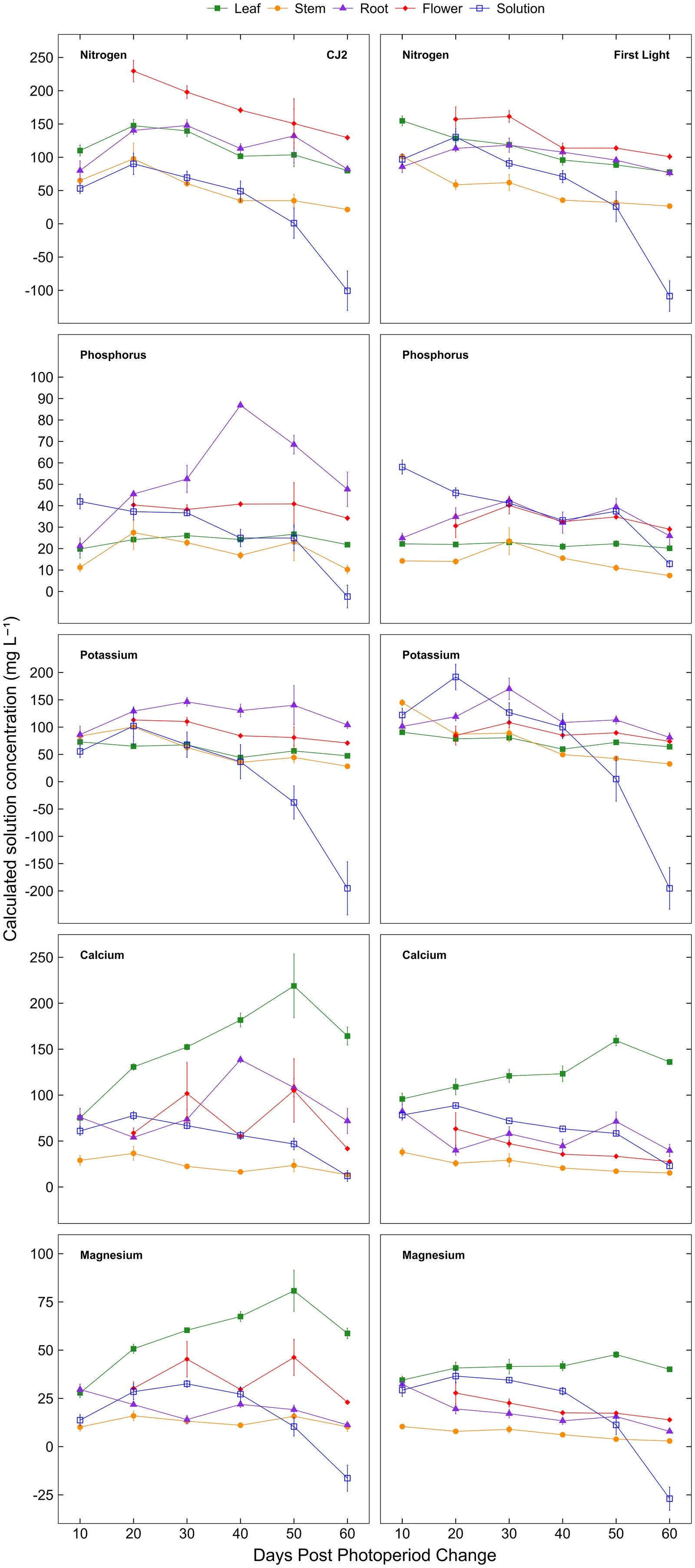
The organ specific calculated nutrient solution input concentrations for nitrogen, phosphorus, potassium, calcium, and magnesium. Each organ per cultivar, CJ2 (left panels) and First Light (right panels) was compared to the solution nutrient removal (input concentration – leachate solution concentration). Green lines and solid squares illustrate the calculated solution input concentration (mg L^-1^) for leaf estimates; whereas, orange lines and solid circles illustrate stem estimates; purple lines and solid triangles illustrate roots estimates; red lines and solid diamonds illustrate flowers estimates; blue lines and open squares illustrate element net removal from solution from days following photoperiod change 10 – 60. Predicted values were calculated on an organ basis using the biomass and water use efficiency observations following Langenfeld et al. (2022) **Equation 1**. Symbols are the means of n = 3 replicates ± SE.

For nitrogen, leaf, flower, and root tissue concentrations generally overestimated net solution removal in CJ2, whereas stem tissue provided the closest estimates throughout most of the reproductive phase. Stem tissue predicted CJ2 nitrogen replenishment with an APD of 7.52%, although it underestimated removal during the final two collection intervals. In First Light, stem tissue also provided the closest estimates during the early reproductive phase before underestimating nitrogen removal at later harvests, while leaf, root, and flower tissues generally overestimated net nutrient removal. Overall, root tissue provided the lowest observed APD prediction of nitrogen replenishment in First Light with an APD of 2.32%.

For phosphorus, leaf and stem tissue concentrations generally underestimated net solution removal in both cultivars. Root tissue tended to overestimate phosphorus removal in CJ2, whereas flower tissue most closely represented phosphorus removal in First Light. Flower tissue provided the lowest observed APD prediction of phosphorus replenishment in CJ2, with an APD of 10.42%.

Potassium predictions derived from root and flower tissue generally overestimated net solution removal in CJ2. In contrast, potassium removal in First Light was generally underestimated by all organs except root tissue during DPP 30 and 40. Stem tissue provided the lowest observed APD prediction of potassium replenishment in CJ2 (APD = 9.66%), whereas root tissue provided the lowest observed APD prediction in First Light (APD = 14.53%).

Leaf tissue consistently overestimated calcium replenishment requirements, with the magnitude of overestimation increasing throughout the reproductive phase, particularly in CJ2. Stem tissue consistently underestimated calcium removal in both cultivars. Although root and flower tissues generally produced closer estimates than leaves or stems, neither organ consistently represented calcium removal throughout the reproductive phase.

Leaf tissue also consistently overestimated magnesium replenishment requirements, whereas stem tissue consistently underestimated magnesium removal. Prior to the decline in magnesium removal after DPP 50, flower tissue provided the closest estimates of magnesium replenishment. Root tissue generally underestimated magnesium removal in both cultivars until the reduction in net solution removal after DPP 40.

#### 3.4.2 Micronutrients

**Figure 7** illustrates the predicted micronutrient replenishment concentrations (Fe, Cu, B, Mn, and Zn) derived from organ-specific tissue concentrations compared with measured net solution removal. Except for B, micronutrient replenishment predictions generally exhibited closer agreement with measured net solution removal than did the macronutrient predictions.

**Figure 7:**
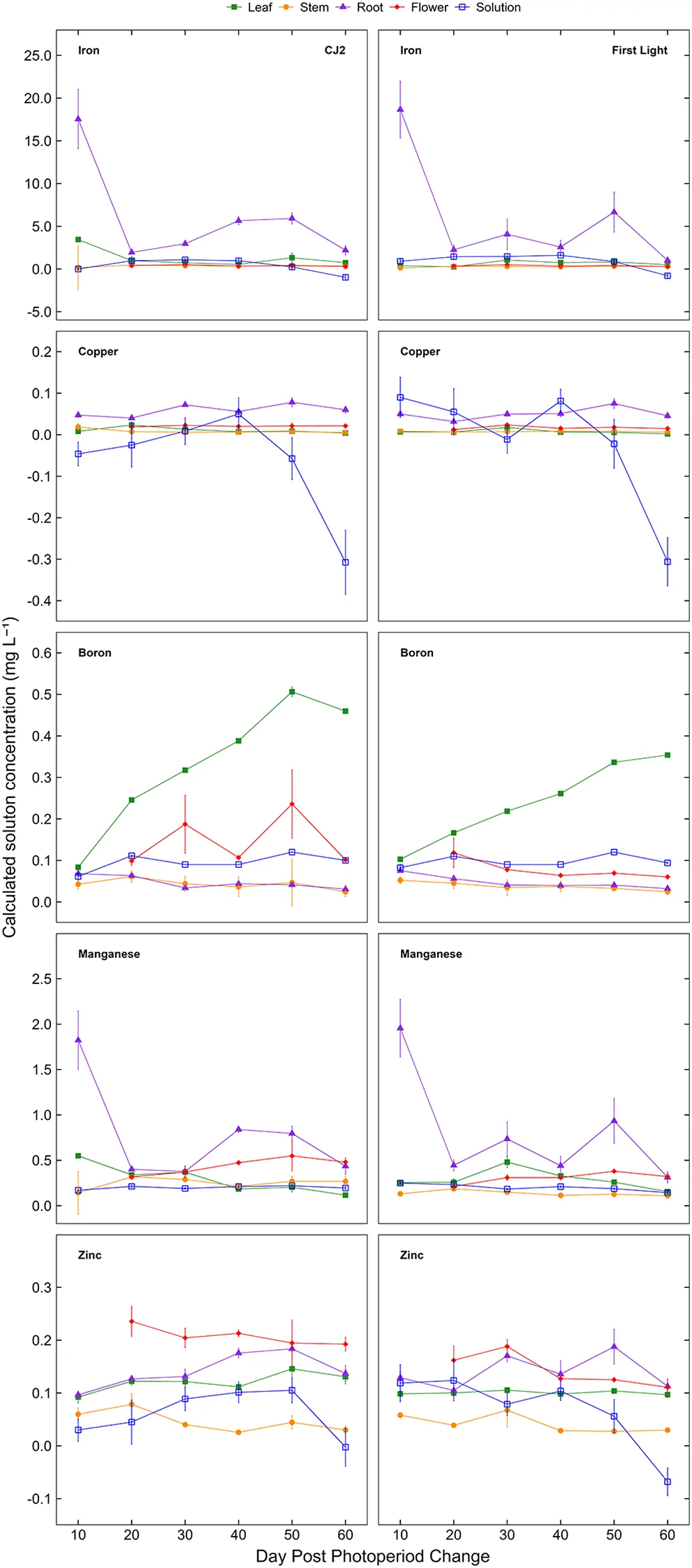
The organ specific calculated nutrient solution input concentration for iron, copper, boron, manganese, and zinc. Each organ per cultivar, CJ2 (left panels) and First Light (right panels), was compared to the solution nutrient removal (input concentration – leachate solution concentration). Green lines and solid squares illustrate the calculated solution input concentration (mg L^-1^) for leaf estimates; whereas, orange lines and solid circles illustrate stem estimates; purple lines and solid triangles illustrate roots; red lines and solid diamonds illustrate flowers; blue lines and open squares illustrate element net removal from solution from days following photoperiod change 10 – 60. Predicted values were calculated on an organ basis using the biomass and water use efficiency observations following Langenfeld et al. (2022) **Equation 1**. Symbols are the means of n = 3 replicates ± SE.

Iron replenishment predictions varied among organs and cultivars throughout the reproductive phase. Root tissue consistently overestimated iron removal in both cultivars. Flower tissue provided the lowest observed organ-specific APD for iron replenishment in CJ2 (48.22%), followed by stem tissue (53.24%).

Copper replenishment predictions differed between cultivars. Organ-specific tissue concentrations generally overestimated copper replenishment requirements in CJ2, whereas predictions for First Light generally underestimated copper removal except at DPP 30, when all organs slightly overestimated replenishment. Root tissue provided the closest prediction of copper replenishment in First Light, with an APD of 6.08%.

Boron tissue-based replenishment predictions exhibited greater deviation than the remaining micronutrients. Leaf tissue consistently overestimated boron replenishment in both cultivars, with prediction error increasing as leaf boron concentrations increased throughout the reproductive phase. Stem and root tissue predictions remained comparatively closer to measured net solution removal.

For manganese and zinc, most organ-specific tissue concentrations overestimated measured net solution removal in both cultivars. Stem tissue provided the lowest observed organ-specific APD for manganese replenishment in CJ2 (27.60%), whereas leaf tissue provided the lowest observed organ-specific APD in First Light (32.04%). Zinc net solution removal remained relatively stable throughout the reproductive phase until the final collection at DPP 60. Organ-specific predictions for zinc generally underestimated replenishment requirements in First Light, whereas overestimation predominated in CJ2.

### 3.5 Weighting organ predictions

**Figure 8** and **Figure 9** compare biomass-weighted replenishment predictors for the macronutrients (**Figure 8**) and micronutrients (**Figure 9**) with measured net solution removal. Biomass-weighted means were calculated using all organs (W) and with flower tissue excluded (WNF).

**Figure 8:**
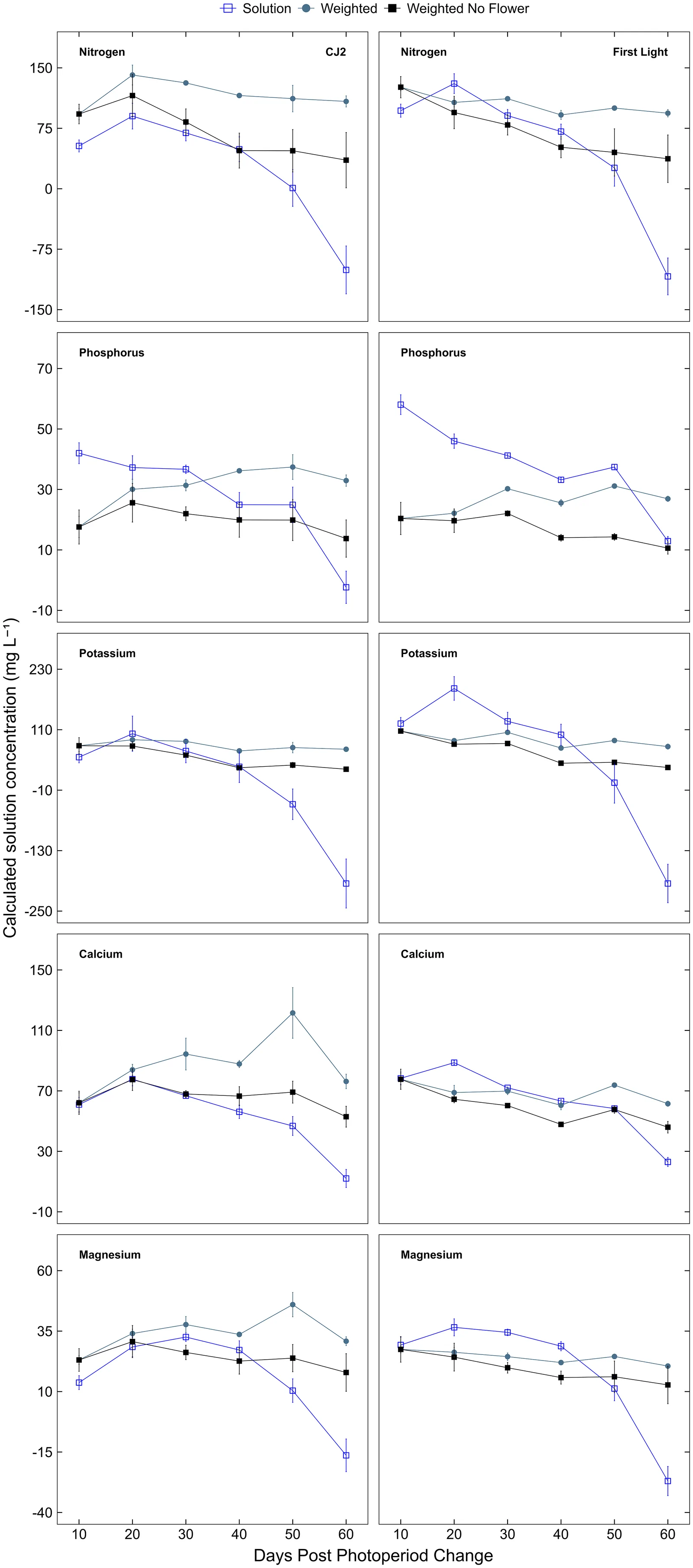
Calculated nutrient solution input concentrations (mg L^-1^) for nitrogen, phosphorus, potassium, calcium, and magnesium expressed as an organ biomass-weighted mean with (W) and without (WNF) flower tissue included in the organ biomass-weighted mean. All organ W values (teal solid circle) along with WNF (black solid squares) per cultivar, CJ2 (left panels) and First Light (right panels), compared to the net solution nutrient removal (open blue squares; input concentration – leachate solution concentration) from days following photoperiod change 10 – 60. Predicted values were calculated using the biomass and water use efficiency observations following Langenfeld et al. (2022), **Equation 1**. Symbols are the means of n = 3 replicates ± SE.

**Figure 9:**
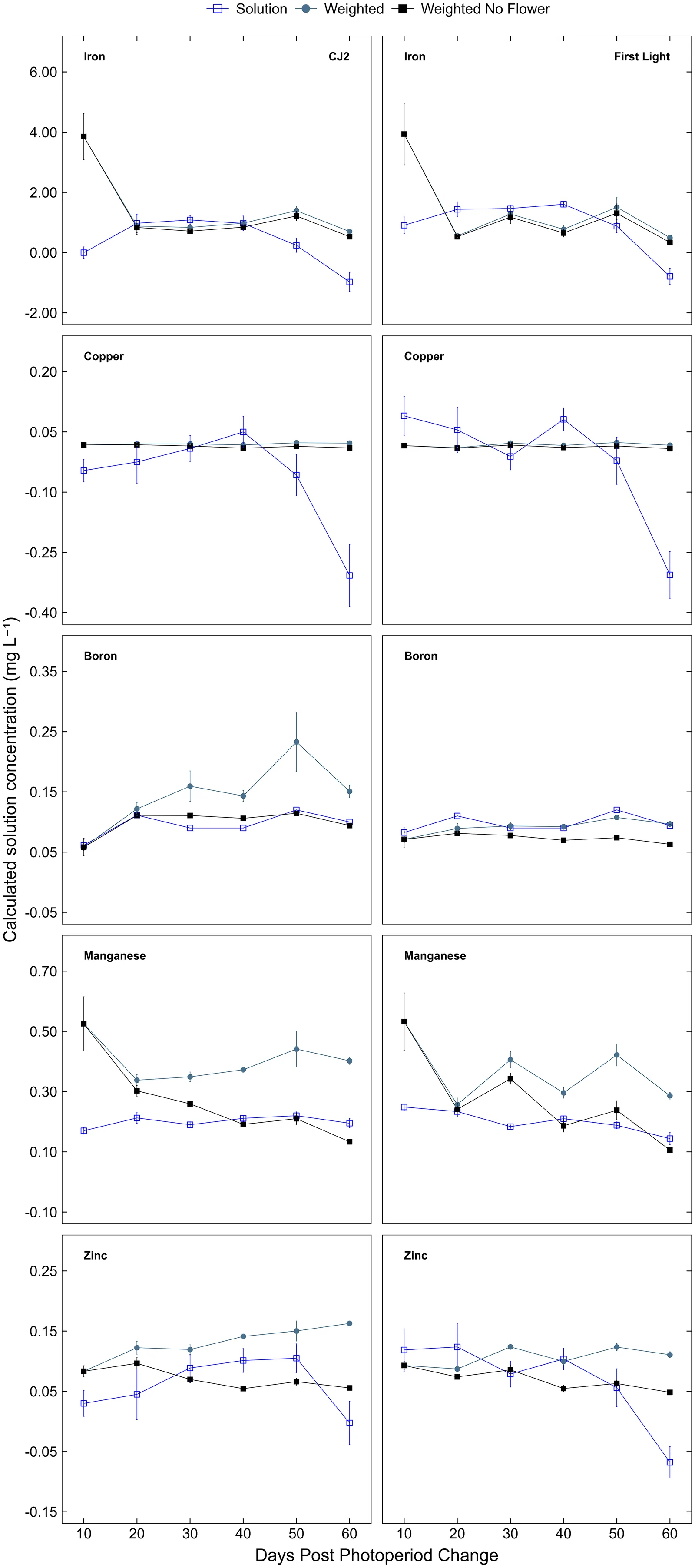
The calculated nutrient solution input concentrations (mg L^-1^) for iron, copper, boron, manganese, and zinc expressed on an organ biomass-weighted mean with (W) and without (WNF) flower tissue included in the organ biomass-weighted mean. All organ W values (teal solid circle) along with WNF (black solid squares) per cultivar, CJ2 (left panels) and First Light (right panels), compared to the net solution nutrient removal (open blue squares; input concentration – leachate solution concentration) from days following photoperiod change 10 – 60. Organ biomass-weighted mean predicted values were calculated using the biomass and water use efficiency observations method following Langenfeld et al. (2022), **Equation 1**. Symbols are the means of n = 3 replicates ± SE. Solution-derived B values are presented for descriptive purposes only.

For cultivar CJ2, WNF provided the lowest APD for N, Ca, Mg, and Zn, with APDs of 7.33%, 0.92%, 5.62%, and 7.15%, respectively. For these elements, WNF either matched or improved upon the lowest observed APD single-organ predictor. Inclusion of flower tissue W did not produce the lowest APD for any element in CJ2.

In First Light, the performance of biomass-weighted means differed among elements. WNF provided the lowest APD for Fe (2.28%), whereas W produced the lowest APDs for Ca, Mg, and Zn, with values of 9.11%, 25.00%, and 0.06%, respectively. For the remaining elements, single-organ predictors produced lower APDs than either biomass-weighted approach.

Overall, the performance of biomass-weighted predictors was dependent on both cultivar and element (**Supplementary Table 4**). WNF most frequently produced the lowest APDs in CJ2, whereas W more frequently produced the lowest APDs in First Light.

### 3.6 Temporal stability of optimum predictors across reproductive development

**Figure 10** compares the harvest-by-harvest performance of individual organ and biomass-weighted replenishment predictors using APD. The identity of the lowest-APD predictor varied throughout the reproductive phase, and no single organ consistently minimized prediction error across all harvests. Single-organ predictors, particularly stem tissue, frequently produced the lowest APD at individual collection intervals but exhibited considerable temporal variability. In contrast, the biomass-weighted predictor WNF maintained comparatively stable APDs throughout the reproductive phase and did not exhibit the pronounced harvest-to-harvest variability observed in the single-organ predictors. Although WNF was not the lowest-APD predictor at every collection interval, its performance remained comparatively consistent across developmental stages (**Figure 10**).

**Figure 10:**
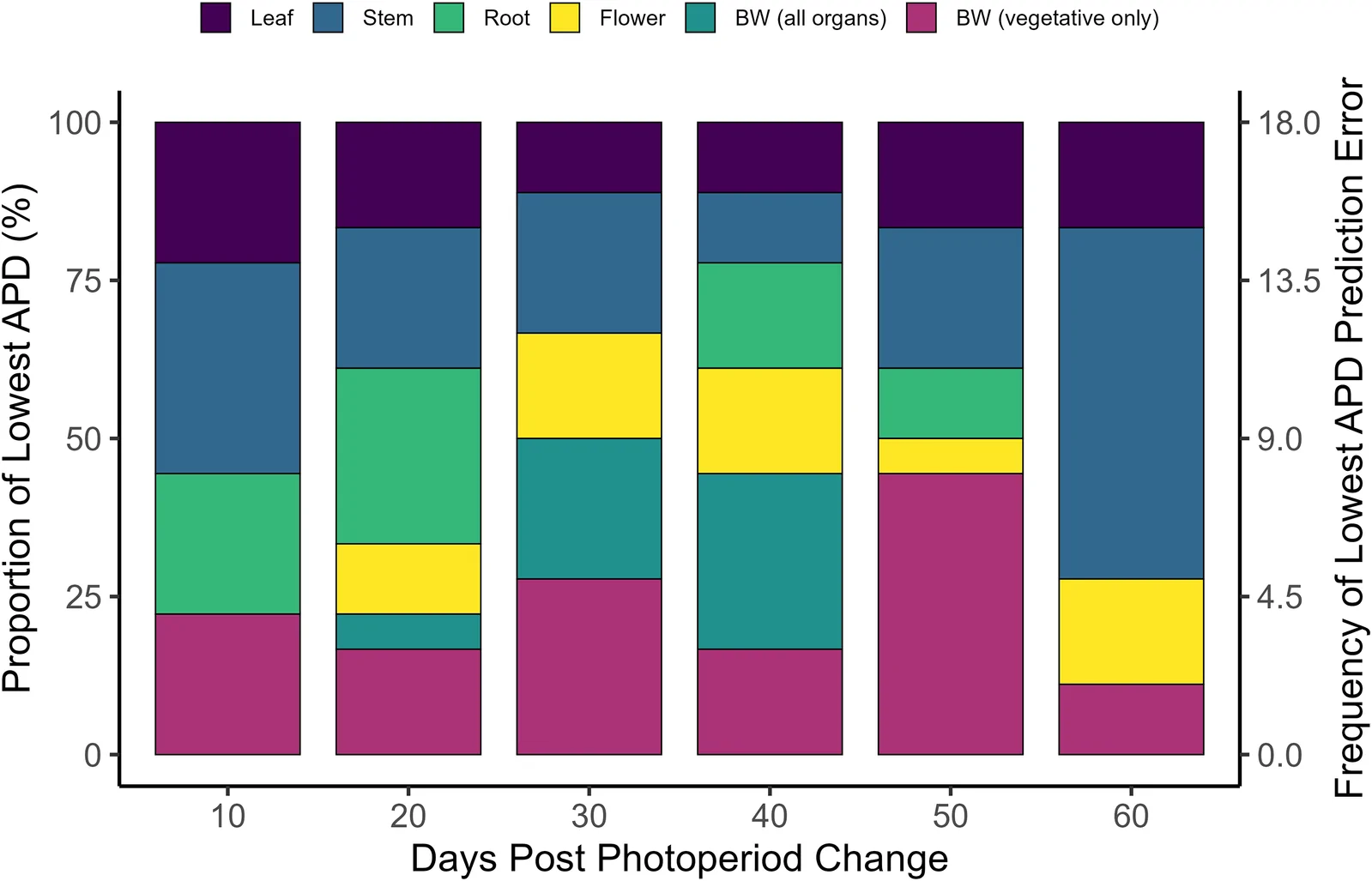
Lowest observed APD (absolute percent deviation) predictor frequency by harvest date calculated between predicted nutrient input and measured nutrient removal from solution. Stacked bars represent the number of element × cultivar combinations for which each predictor produced the lowest APD at a given harvest. Predictor identity varied across developmental stages, with no single organ consistently minimizing error through time. BW, biomass-weighted composite predictor. All organs = leaf + stem + root + inflorescence; vegetative only = leaf + stem + root. Note, B APD is not included in the figure.

### 3.7 Optimal replenishment prediction method

To summarize predictor performance across the reproductive phase, each candidate replenishment predictor, including individual organ concentrations and biomass-weighted means, was averaged over the interval where measured net solution removal provided the most reliable benchmark. **Supplementary Figures 1** and **2** compare these mean predicted replenishment concentrations with the corresponding mean measured net solution removal for each predictor–element combination. The predictor with the lowest APD for each cultivar–element combination is presented to illustrate systematic prediction bias, where values above the measured net solution removal indicate overprediction and values below indicate underprediction. The summarized comparisons include only the harvest interval used for APD analysis. The initial harvest was excluded because tissue and leachate measurements were not directly paired, whereas DPP 50 and 60 were excluded because increasing substrate nutrient concentrations deviated from mass-balance estimates. Across the macronutrients, biomass-weighted predictors produced the lowest APD in most cultivar–element comparisons (**Supplementary Figure 1**). Similar trends were observed for the micronutrients, where biomass-weighted predictor WNF produced the lowest APD in five of the ten cultivar–element comparisons (**Supplementary Figure 2**).

## 4 Discussion

### 4.1 Transpiration-driven mass balance during the reproductive phase

The present findings extend vegetative-phase mass-balance principles into the reproductive phase of *C. sativa*, demonstrating that net solution removal from solution remains tightly coupled to transpiration-driven fluxes rather than fixed solution concentrations (Langenfeld et al., 2022; Langenfeld and Bugbee, 2024; Powell and Bauerle, 2026). Across both cultivars, nutrient extraction from the irrigation solution increased following floral initiation, peaked mid-flowering, and declined steadily thereafter (**Figures 1**, **2**), mirroring concurrent changes in WUE (**Figure 3**). This temporal coupling confirms that nutrient demand during flowering is dynamic and coupled to whole-plant water flux.

Consistent with prior zero-discharge and closed-loop studies, nutrient removal from solution tracked plant uptake rather than remaining proportional to imposed fertigation setpoints (Langenfeld et al., 2022; Langenfeld and Bugbee, 2024). Declining removal later in the generative phase coincided with reduced transpiration and WUE, underscoring the biophysical basis of transpiration-scaled nutrient delivery. Together, these results highlight a fundamental limitation of static EC setpoints during reproductive development, when nutrient demand decouples from imposed fertigation targets as transpiration declines and inflorescences mature.

### 4.2 Organ divergence and phenology-driven partitioning

Organ-level tissue analysis revealed pronounced divergence in elemental concentrations across flowering. Leaf Ca and Mg concentrations increased steadily during reproduction, while leaf N declined, consistent with reduced vegetative expansion and altered source–sink relations (Saloner and Bernstein, 2023; Massuela et al., 2023). These shifts intensified as phenology advanced, undermining the assumption that single-organ elemental concentrations reliably reflect whole-plant nutrient demand throughout flowering.

Although Mg net solution removal from solution declined over time (**Figure 1**), Mg concentrations in leaf tissue continued to increase, particularly in CJ2. Because tissue harvests were conducted on pooled leaf biomass rather than selectively sampling upper, recently expanded leaves, this pattern indicates progressive internal redistribution of Mg from stems and roots to leaves rather than sustained external acquisition. Magnesium is phloem mobile, and its remobilization from structural tissues to metabolically active leaves under declining growth and transpiration aligns with established models of nutrient recycling during reproductive development and senescence (Marschner, 2012). Correspondingly, decreasing Mg pools in stems and roots support the interpretation that internal translocation governed leaf Mg accumulation late in flowering.

Interestingly, Ca, despite being classically regarded as phloem immobile, exhibited a qualitatively similar temporal trajectory in leaf tissue accumulation. While absolute Ca concentrations differed from Mg, the shapes of the nutrient removal and tissue accumulation curves were similar (**Figure 4**). This convergence likely reflects shared biophysical constraints imposed by declining transpiration and growth rates during late flowering, which limit xylem delivery to expanding tissues while concentrating Ca in persistent leaves as biomass accumulation slows (Hawkesford et al., 2012). Thus, parallel Ca and Mg trends do not imply shared mobility mechanisms, but rather a common response to phenology-driven reductions in water flux.

Collectively, these organ-specific reproductive stage trajectories demonstrate that internal redistribution and phenological context influence tissue nutrient profiles during flowering. Reliance on single-organ diagnostics during the reproductive phase therefore risks systematic misrepresentation of whole-plant nutrient removal from solution, with both the direction and magnitude of bias dependent on element mobility and developmental stage. Similar patterns of organ divergence have been reported in flowering *C. sativa* and other reproductive crops, where nutrient partitioning shifts rapidly with sink dominance (Westmoreland and Bugbee, 2022; Saloner and Bernstein, 2023).

### 4.3 Biomass-weighted nutrient replacement predictors and genotype-specific responses

Integrating organ nutrient concentrations using biomass-weighted means generally provided a more accurate representation of whole-plant nutrient removal from solution than single-organ replacement estimates across most elements and time points (**Figures 8**, **9**). Biomass-weighted means excluding flower tissue in general aligned consistently with measured solution removal for N, Ca, Mg, B, and Zn in CJ2, and for Fe in First Light. This outcome supports exclusion of reproductive tissues when estimating nutrient replenishment amounts, given recent evidence for *C. sativa* luxury accumulation and nutrient storage in flowers without proportional yield or quality benefits (Hershkowitz et al., 2025; Westmoreland and Bugbee, 2022).

The correspondence between declining solution removal of N and K and decreasing predicted replenishment concentrations further validates the transpiration-driven nutrient replenishment mass-balance framework. As flowering progressed and plants approached senescence, reductions in transpiration and WUE coincided with diminished drawdown from solution of these highly mobile elements, consistent with declining physiological age organ metabolic activity in *C. sativa* (Bauerle et al., 2020). Predicted replacement inputs decreased accordingly, confirming that the framework responds to phenology-driven reductions in nutrient demand. However, we note that the mass-balance nutrient replenishment framework consistently over estimated replenishment rates during this phase of the regenerative cycle.

In contrast, Mg displayed an opposing pattern in CJ2, where predicted replenishment input concentrations increased despite declining removal from solution. This apparent discrepancy could be a result of internal redistribution where increasing leaf Mg concentrations reflect translocation from stems and roots rather than increased root acquisition. When predicted replenishment rates are derived from biomass-weighted tissue stoichiometry scaled by whole-plant water use, the framework captures changes in internal elemental allocation. Thus, increasing predicted Mg replenishment amounts do not imply increased Mg net removal from the solution, but rather could reflect shifting internal pools under declining transpiration.

Some genotype-specific differences were evident throughout the reproductive phase. First Light removed greater quantities of nutrients early in flowering, whereas CJ2 maintained higher WUE, producing distinct mass-balance replenishment amounts under identical fertigation regimes. These differences are consistent with genotype-dependent coupling between transpiration, nutrient flux, and canopy function previously reported in hemp and cannabis systems (Baumgardner et al., 2021; Tang et al., 2018; Saloner et al., 2019; Powell and Bauerle, 2026). Although individual organs occasionally approximated measured net solution removal better than WNF for specific elements, these relationships were transient and element-specific. Overall, biomass-weighted predictors provided the most consistent and physiologically grounded means to estimate whole-plant nutrient replenishment amounts during the flowering phase in *C. sativa*.

### 4.4 Temporal robustness and model applicability

While mean APD comparisons identified element- and cultivar-specific lowest observed APD replenishment predictors, the harvest-resolved analysis revealed that predictor identity was not constant across reproductive development. Single-organ replenishment predictors, particularly stems and roots, occasionally minimized prediction error but exhibited pronounced temporal variability. This instability in one particular organ or biomass-weighted input tissue element concentration could reflect developmental shifts in organ function, biomass allocation, and internal nutrient redistribution, which alter the relationship between local tissue concentration and whole-plant nutrient demand during reproductive development (Saloner and Bernstein, 2023; Massuela et al., 2023). Consequently, tissue element concentration predictors that perform well at a single developmental stage may systematically misrepresent demand when applied across the entire reproductive cycle.

In contrast, biomass-weighted composite predictors, especially those that excluded reproductive tissue, demonstrated greater temporal stability, which comparatively resulted in lower error across the reproductive phase (**Supplementary Figures 1**, **2**). The exclusion of reproductive tissue likely reduced over estimation that could result from luxury accumulation and transient nutrient storage that does not scale proportionally with transpiration-driven uptake (Westmoreland and Bugbee, 2022; Hershkowitz et al., 2025). From a fertigation perspective, temporal robustness is more operationally meaningful than intermittent optimality. Nutrient replacement decisions occur continuously rather than at individual developmental stages, particularly in controlled-environment production systems where fertigation schedules can alter throughout the production cycle (Langenfeld et al., 2022; Sperling et al., 2020). A nutrient replacement predictor that minimizes cumulative error across time therefore provides a more reliable estimate for dynamic nutrient management throughout a crop’s life cycle.

These findings reinforce that reproductive-phase nutrition cannot be inferred from single-organ diagnostics or static tissue element concentration targets. Instead, accurate nutrient replacement requires integration of biomass partitioning, phenological stage, and transpiration-scaled flux (Langenfeld et al., 2022; Langenfeld and Bugbee, 2024). The temporal instability of organ-specific predictors further underscores why fixed EC or input concentration setpoints fail during flowering: as internal nutrient allocation and water flux decline, the relationship between tissue concentration and net nutrient removal from solution becomes increasingly nonlinear (Saloner and Bernstein, 2023; Massuela et al., 2023). Organ biomass-weighted mass-balance approaches mitigate this individual organ estimate instability by integrating organ-level divergence across the whole plant into a single physiologically grounded predictor of whole-plant demand (Powell and Bauerle, 2026).

### 4.5 Temporal dynamics and the failure of static nutrient input setpoints

The temporal variation of net nutrient removal from solution across the reproductive phase in *C. sativa* illustrates why static fertigation setpoints underperform during *C. sativa* reproductive development. Early flower development coincided with strong inflorescence sink activity, elevated transpiration, and pronounced solution drawdown, whereas late-stage flowering was characterized by declining net solution removal despite continued nutrient availability (**Figures 1**, **2**). This downturn tracked reductions in transpiration and altered organ nutrient allocation, reinforcing the need for stage-specific adjustments in nutrient delivery (Langenfeld et al., 2022; Saloner and Bernstein, 2023).

Importantly, declining net solution removal from solution does not imply uniform depletion of tissue nutrient pools. For mobile elements such as N and K, reduced nutrient solution drawdown was accompanied by declining tissue concentrations, consistent with diminished ontogenetic organ metabolic activity and progressive nutrient remobilization as plants approached senescence (Bauerle et al., 2020). In these cases, decreasing predicted replacement concentrations aligned with both solution- and tissue-based indicators of nutrient concentration.

By contrast, Mg exhibited a temporal decoupling between net solution removal and tissue accumulation, particularly in CJ2. Although Mg removal from solution declined, leaf Mg concentrations continued to increase due to internal redistribution from stems and roots. Under these conditions, static fertigation setpoints would misinterpret continued tissue accumulation as sustained tissue external demand, whereas the mass-balance framework, when paired with organ-level tissue analysis, correctly distinguishes internal translocation from root uptake.

Taken together, organ-level nutrient concentration divergence, plant internal nutrient redistribution, and declining transpiration across the reproductive phase demonstrate that reproductive-phase nutrient requirements in *C. sativa* is governed by dynamic, organ metabolic activity and water flux rather than static nutrient input solution concentrations, requiring fertigation strategies that integrate phenology, biomass partitioning, and transpiration-scaled mass balance to accurately reflect whole-plant nutrient replenishment.

### 4.6 Nutrient replenishment versus requirement

The present study estimated reproductive-stage nutrient replenishment targets for *C. sativa* by integrating organ-specific tissue nutrient concentrations, organ biomass-weighted means, WUE, and measured net solution removal within a transpiration-driven mass-balance framework. Perlite was selected to provide an aerated rooting medium with limited cation-exchange buffering, thereby reducing, but not eliminating, substrate contributions to changes in solution nutrient concentrations. This choice does not imply superiority over stone wool or expanded clay, which were not evaluated experimentally. Higher-CEC substrates, such as coir, would require explicit consideration of exchange-related nutrient storage and release in the nutrient balance. Recently, Langenfeld and Bugbee (2026) quantified optimal nutrient requirements for three lettuce cultivars using a zero-discharge hydroponic system, providing a useful benchmark for comparison. Therefore, the values reported here should be interpreted as operational replenishment estimates rather than definitive physiological nutrient requirements.

Relative to the lettuce requirement values, average *C. sativa* replenishment estimates were lower for N, K, Cu, and Zn, higher for P, Ca, Mg, and Fe, and similar for Mn. This element-specific pattern also held for vegetative-stage *C. sativa*, as reported by Powell and Bauerle (2026), indicating that the divergence between lettuce requirement values and *C. sativa* replenishment estimates was not restricted to reproductive development. These element-specific differences likely reflect species-level stoichiometry, crop developmental stage, organ biomass partitioning, and system-level retention or enrichment processes. Importantly, net solution removal may exceed plant nutrient accumulation because removed nutrients can be partitioned among plant uptake, substrate retention, precipitation, microbial immobilization, or other root-zone sinks. Thus, higher replenishment estimates for elements such as P, Ca, Mg, or Fe should not be interpreted automatically as evidence of greater physiological requirement or excessive fertilization. From a nutrient management perspective, this distinction is important because elemental concentrations supplied above measured net solution removal are unlikely to improve crop nutrition under comparable conditions. Surplus nutrients must either accumulate in the root-zone solution, be retained within the substrate/root-zone system or exit the container as nutrient-rich leachate. Accordingly, measured net solution removal, when paired with mass-balance-derived replenishment estimates, provides an operational benchmark for identifying fertilizer inputs that exceed crop demand, potentially decreasing input costs and nutrient-discharge risk.

The crop comparison is also constrained by developmental context. Lettuce is a short-cycle vegetative crop, whereas reproductive *C. sativa* develops strong floral sinks, changing root relationships, and phenology-dependent nutrient redistribution. Direct numerical comparison between these systems should therefore be interpreted cautiously. The comparison nevertheless reinforces the broader principle that mass-balance fertigation must distinguish between crop nutrient requirement, solution replenishment, and system-level nutrient removal. Thus, the value of the mass-balance approach is not that it produces a universal nutrient recipe, but that it provides a quantitative framework for distinguishing biological requirements from operational replenishment across crops, cultivars, developmental stages, and production systems.

### 4.7 Implications for fertigation practice and policy

Across controlled-environment agriculture (CEA), evidence increasingly supports transpiration-coupled, mass-balance fertigation strategies over fixed nutrient recipes (Langenfeld et al., 2022; Sperling et al., 2020; Powell and Bauerle, 2026). In greenhouse and CEA systems, nutrient replenishment inputs scaled to water loss consistently reduce discharge and improve nutrient use efficiency (Blok et al., 2023; Li et al., 2024; Westmoreland and Bugbee, 2022). Dynamic fertigation studies in tomato further demonstrate large discrepancies between balance-based uptake estimates and dry-matter accounting, revealing system-level sinks that static EC management fails to detect (Cedeño et al., 2023, 2024). Despite decades of fertigation research in horticultural crops, most nutrient solution input concentration management frameworks continue to rely on static solution concentrations or EC targets that cannot resolve phenology-driven shifts in transpiration, internal nutrient redistribution, or the decoupling of tissue accumulation from external uptake observed during reproductive development.

In *C. sativa*, fertigation strategy reshapes the ionome along with downstream biomass and cannabinoid outcomes, emphasizing that nutrient flux—not solution concentration alone—governs crop performance (Velechovský et al., 2024). Operationally, nutrient mass-balance frameworks can reduce fertilizer loss, minimize wastewater handling, and facilitate compliance with emerging regulatory constraints that prioritize measurement-based nutrient stewardship (Ontario Ministry of the Environment, Conservation and Parks (MECP), 2022). Because EC integrates all ions into a single signal and obscures element-specific behavior—such as K-induced suppression of Ca and Mg uptake or redistribution-driven tissue accumulation—flux-based fertigation provides a more biophysical and mass conservation defensible basis for specifying elemental resupply per unit transpired water (Bugbee, 2004; Langenfeld and Bugbee, 2024; Langenfeld et al., 2022; Sperling et al., 2020; Powell and Bauerle, 2026).

## 5 Conclusion

This study quantified net nutrient removal from solution, organ-level and biomass-weighted elemental composition, and water use across the reproductive phase of *C. sativa*, demonstrating that net nutrient removal from solution declines with reduced transpiration late in the flowering stage. Organ nutrient concentrations diverged as phenology advanced: leaves accumulated Ca and Mg despite declining net nutrient removal from solution, while N declined; roots and stems diverged for K, consistent with *C. sativa* reproductive sink dominance and internal nutrient remobilization (Saloner and Bernstein, 2023).

Biomass-weighted means of non-reproductive organs most accurately tracked solution removal over time, with element, organ and cultivar-specific exceptions (e.g., N approximated by stems in CJ2 and roots in First Light). Cultivar contrasts were observed in this study, however, this variation was marginal: First Light removed more nutrients early in flowering, whereas CJ2 maintained higher WUE, indicating that replenishment strategies may be genotype specific. The most accurate predictor shifted with phenology, reinforcing that static nutrient proxies are insufficient during flowering and that fertigation should at least respond to observed solution drawdown rather than maintain fixed input targets (Westmoreland and Bugbee, 2022; Powell and Bauerle, 2026).

Collectively, these results support a transpiration-scaled, nutrient and WUE mass-balance framework for reproductive-phase fertigation, aligning with critiques of EC-centric control and advancing contemporary zero-discharge nutrient management strategies (Langenfeld et al., 2022; Langenfeld and Bugbee, 2024).

## Data Availability

The raw data supporting the conclusions of this article will be made available by the authors, without undue reservation.

## Supplementary material

Supplementary Figure 1 The mean predicted replenishment concentration and mean measured net solution removal across the DPP 10–40 four harvest intervals for cultivars CJ2 (left panel) and First Light (right panel) (mg L^−1^) for macro elements nitrogen, phosphorus, potassium, calcium and magnesium. Weighted value (teal solid circle); weighted no flower (black solid square); stem (orange solid circle); flower (red solid diamond); root (purple solid triangle) and solution nutrient removal (input concentration – leachate nutrient concentration, blue open square) mean of all harvest intervals ± SE (n = 4).

Supplementary Figure 2 The mean predicted replenishment concentration and mean measured net solution removal across the DPP 10–40 four harvest intervals for cultivars CJ2 (Left) and First Light (Right) (mg L^−1^) for micro elements copper, iron, manganese, and zinc. Weighted value (teal solid circle); weighted no flower (black solid square); leaf (green solid square); stem (orange solid circle); root (purple solid triangle) and solution nutrient removal (input concentration – leachate nutrient concentration, blue open square) mean of all harvest intervals ± SE (n = 4).
