# Supplementary material for: Predicting reproductive-phase nutrient replenishment in Cannabis sativa L. via a transpiration-driven mass-balance framework
Source: Front Plant Sci. 2026 Oct 12;17:1955837. doi: 10.3389/fpls.2026.1955837 (PMC13711131; doi:10.3389/fpls.2026.1955837)
Supplement: Supplementary file 1 [file Table1.docx]

Supplementary Table 1. Interpretation at unadjusted α = 0.05: Significant cultivar main effects were detected for P, K, Ca, and Fe (p ≤ 0.05), whereas significant date x cultivar interactions were detected for Mg and Mn (p ≤ 0.05). B is excluded because leachate B concentrations were below the method-specific limit of quantification.

| **Element** | **Date F (df)** | **p** | **Cultivar F (df)** | **p** | **Date × Cultivar F (df)** | **p** |
| --- | --- | --- | --- | --- | --- | --- |
| N | 47.753 (6, 71.636) | <0.0001 | 4.139 (1, 15.234) | 0.05969 | 0.868 (6, 71.636) | 0.52301 |
| P | 69.500 (6, 70.999) | <0.0001 | 11.879 (1, 14.839) | 0.003647 | 2.189 (6, 70.999) | 0.053995 |
| K | 34.286 (6, 71.528) | <0.0001 | 5.589 (1, 15.132) | 0.03186 | 0.822 (6, 71.528) | 0.55630 |
| Ca | 128.700 (6, 70.893) | <0.0001 | 7.265 (1, 14.715) | 0.01683 | 1.338 (6, 70.893) | 0.25178 |
| Mg | 56.150 (6, 71.608) | <0.0001 | 1.287 (1, 15.187) | 0.27420 | 2.540 (6, 71.608) | 0.02755 |
| Fe | 28.783 (6, 71.552) | <0.0001 | 8.031 (1, 15.136) | 0.01248 | 0.840 (6, 71.552) | 0.54327 |
| Cu | 13.498 (6, 71.395) | 3.612×10⁻10 | 2.210 (1, 14.760) | 0.1581 | 0.833 (6, 71.395) | 0.5487 |
| Mn | 20.694 (6, 70.303) | <0.0001 | 4.256 (1, 12.386) | 0.06072 | 7.617 (6, 70.303) | <0.00001 |
| Zn | 4.210 (6, 71.290) | 0.001109 | 0.526 (1, 14.489) | 0.479873 | 1.626 (6, 71.290) | 0.152542 |

Supplementary Table 2. The Bonferroni adjusted p-value across the seven date-specific cultivar contrasts within each element. B is excluded because leachate B concentrations were below the method-specific limit of quantification.

| **Element** | **Significant DPP** | **Direction / estimate** | **Bonferroni-adjusted p** |
| --- | --- | --- | --- |
| N | None | — | — |
| P | DPP 10 | First Light > CJ2 by ~16.06 ppm | 0.0019 |
| P | DPP 60 | First Light > CJ2 by ~16.11 ppm | 0.0138 |
| K | None | — | — |
| Ca | DPP 10 | First Light > CJ2 by 17.36 ppm | 0.0062 |
| Mg | DPP 10 | First Light > CJ2 by 15.57 ppm | 0.0110 |
| Fe | DPP 10 | First Light > CJ2 by 0.909 ppm | 0.0225 |
| Cu | None | — | — |
| Mn | DPP 0 | First Light > CJ2 by 0.11125 ppm | <0.0001 |
| Mn | DPP 10 | First Light > CJ2 by 0.07875 ppm | 0.0007 |
| Zn | None | — | — |

Supplementary Table 3. Tissue-based predictor observations and measured net solution removal observations were independently resampled with a 10,000-iteration nonparametric bootstrap analysis. Within cultivar × harvest, and APD was recalculated over DPP 10–40. The resulting distributions provide 95% bootstrap intervals and the frequency with which each predictor produces the lowest APD. B is excluded because leachate B concentrations were below the method-specific limit of quantification.

| **Element** | **Cultivar** | **Lowest observed APD predictor** | **Observed APD (%)** | **Bootstrap 95% CI (%)** | **Lowest-APD frequency (%)** |
| --- | --- | --- | --- | --- | --- |
| N | First Light | Root | 9.26 | 0.50–23.86 | 41.0 |
| N | CJ2 | Stem | 1.41 | 0.47–32.07 | 89.2 |
| P | First Light | Root | 24.68 | 15.05–33.19 | 100.0 |
| P | CJ2 | Weighted | 18.25 | 6.64–27.75 | 99.4 |
| K | First Light | Root | 7.73 | 0.44–22.84 | 99.0 |
| K | CJ2 | WNF | 4.52 | 0.74–65.69 | 28.3 |
| Ca | First Light | Weighted | 8.42 | 3.02–13.85 | 99.6 |
| Ca | CJ2 | WNF | 4.73 | 0.21–14.00 | 100.0 |
| Mg | First Light | Weighted | 22.42 | 13.99–29.58 | 50.6 |
| Mg | CJ2 | WNF | 0.62 | 0.21–16.57 | 82.1 |
| Fe | First Light | WNF | 16.18 | 0.89–63.06 | 54.4 |
| Fe | CJ2 | Leaf | 20.53 | 1.33–53.36 | 88.1 |
| Cu | First Light | Root | 15.27 | 1.72–390.01 | 84.9 |
| Cu | CJ2 | Stem | 407.58 | 6.94–1424.17 | 67.0 |
| Mn | First Light | Stem | 19.54 | 12.96–25.46 | 95.3 |
| Mn | CJ2 | Leaf | 32.14 | 18.44–46.95 | 92.5 |
| Zn | First Light | Weighted | 5.04 | 0.53–32.83 | 34.8 |
| Zn | CJ2 | WNF | 14.76 | 0.83–91.55 | 54.4 |

Supplementary Table 4. Prediction error metrics, R², RMSE, MAE, are reported alongside APD to report other prediction-error metrics in addition to APD. B is excluded because leachate B concentrations were below the method-specific limit of quantification.

| **Element** | **Cultivar** | **Lowest APD** | **Lowest RMSE** | **Lowest MAE** | **Highest R²** |
| --- | --- | --- | --- | --- | --- |
| N | First Light | Root | Weighted | Root | WNF (0.281) |
| N | CJ2 | Stem | Stem | Stem | Leaf (0.894) |
| P | First Light | Root | Root | Root | Weighted (0.441) |
| P | CJ2 | Weighted | Leaf | Leaf | Root (0.966) |
| K | First Light | Root | Root | Root | Leaf (0.102) |
| K | CJ2 | WNF | Stem | Stem | Weighted (0.840) |
| Ca | First Light | Weighted | Weighted | Weighted | WNF (0.419) |
| Ca | CJ2 | WNF | WNF | WNF | WNF (0.751) |
| Mg | First Light | Weighted | Weighted | Weighted | Leaf (0.178) |
| Mg | CJ2 | WNF | WNF | WNF | Weighted (0.998) |
| Fe | First Light | WNF | Leaf | Leaf | Stem (0.971) |
| Fe | CJ2 | Leaf | Leaf | Leaf | WNF (0.996) |
| Cu | First Light | Root | Root | Root | Leaf (0.878) |
| Cu | CJ2 | Stem* | Leaf | Stem | WNF (0.898) |
| Mn | First Light | Stem | Stem | Stem | Leaf (0.973) |
| Mn | CJ2 | Leaf | Leaf | Leaf | Stem (0.723) |
| Zn | First Light | Weighted | Leaf | Leaf | Weighted (0.981) |
| Zn | CJ2 | WNF | WNF | WNF | Root (0.759) |

*CJ2 Cu APD is unstable because the measured mean approaches zero. RMSE and MAE remain interpretable. R² is descriptive only because the retained validation window contains four harvest means; it should not be used independently to select the preferred predictor.
